# Acute Intermittent Porphyria With Epilepsy as the Initial Symptom and Posterior Reversible Encephalopathy Syndrome: A Case Report

**DOI:** 10.1155/crnm/5596570

**Published:** 2025-10-23

**Authors:** Wei Li, Zhi-Yun Lian, Xiu-Juan Mi, Jun Tang

**Affiliations:** Department of Neurology, Chongqing Hospital of Traditional Chinese Medicine, Chongqing 400021, China

**Keywords:** acute intermittent porphyria, hydroxymethylbilane synthase, magnetic resonance imaging, posterior reversible encephalopathy syndrome

## Abstract

Acute intermittent porphyria (AIP) is a rare hereditary metabolic disorder, manifesting in a series of neuropsychiatric symptoms and abdominal pain. Posterior reversible encephalopathy syndrome (PRES) is also an uncommon clinical syndrome characterized by localized cerebral edema in the posterior part of the brain, accompanied by abnormal signal changes in white matter areas. Typically, AIP lacks specific clinical symptoms and cranial imaging features, making the diagnosis difficult. In this case, a young male with AIP presented with intermittent abdominal pain prior to epilepsy. Therefore, the diagnosis of AIP should be considered when epilepsy is associated with PRES.

## 1. Introduction

Acute intermittent porphyria (AIP) is a rare inherited metabolic disorder. It is caused by defects in enzymes associated with porphyrin synthesis, in particular reduced activity of hydroxymethylbilane synthase (HMBS) (also referred to as porphobilinogen [PBG] deaminase [PBGD]; OMIM 609806), leading to the accumulation of porphyrin precursors (e.g., PBG and δ-aminolevulinic acid [δ-ALA]) in the body [1]. The mutations associated with AIP have been observed only in the HMBS gene, which encodes HMBS (PBGD). Symptoms of AIP typically manifest during acute attacks and may include severe abdominal cramps, a common initial symptom often leading to misdiagnosis as acute abdominal conditions, as observed in this case where the patient had intermittent abdominal pain without timely medical consultation. Neurological symptoms are also prominent, such as acute peripheral polyneuropathy (evident in the patient's electromyographic findings of neurogenic damage), seizures (consistent with the patient's presentation of unconsciousness and limb twitching), and muscle weakness or atrophy (noted in the supraspinatus, infraspinatus, and deltoid muscles). Psychosis may also occur. The diagnosis and treatment of a young patient with AIP who showed epilepsy and posterior reversible encephalopathy syndrome (PRES) as the first symptoms, who was admitted to our department, are reported. This case is clinically meaningful and unique for two reasons. First, it presents an atypical initial manifestation of AIP: epilepsy, which is rarely reported as the first symptom in AIP patients, who more commonly present with abdominal pain or neuropsychiatric symptoms [1]. This challenges conventional diagnostic paradigms, emphasizing the need to consider AIP in the differential diagnosis of cryptogenic epilepsy, particularly when accompanied by unexplained abdominal pain. Second, the concurrent occurrence of AIP with PRES is exceptionally rare. This unique comorbidity expands our understanding of AIP's neurological complexity. In addition, the case highlights critical pitfalls, such as the use of sodium valproate (an AIP exacerbant), and underscores diagnostic clues such as light-induced wine-colored urine, lessons that can improve clinical practice.

## 2. Case Report

**Figure 1:**
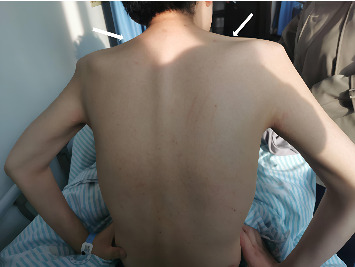
Muscle atrophy observed in the supraspinatus, infraspinatus, and deltoid muscles.

**Figure 2:**
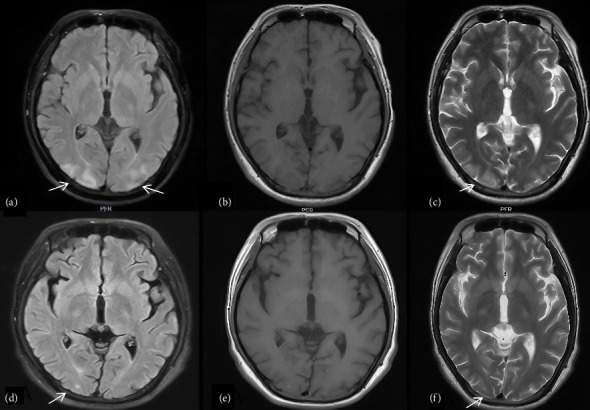
(a) Cranial MRI showed diffuse symmetrical patchy FLAIR with high signals (arrows) in the bilateral parieto-occipital cortex and bilateral cerebellar hemispheres, (b) equal or low signal in T1WI, (c) high signal in T2WI, and (d, e, f) the abnormalities seen in FLAIR, T1WI, and T2WI had essentially disappeared in a cranial MRI performed 18 d later.

**Figure 3:**
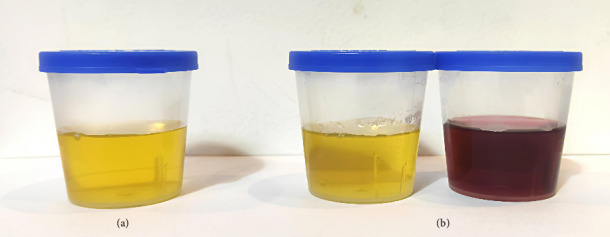
Upon exposure to sunlight, the patient's urine darkens and takes on a wine-colored hue (a to b).

**Figure 4:**
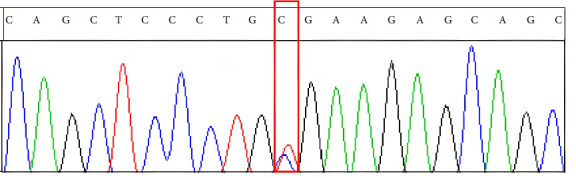
The subjects were found to carry a heterozygous mutation at the c.445C > T site of the HMBS gene, resulting in the termination of translation at Residue 149 of the protein and protein truncation.

The patient was a 28-year-old male who was admitted to our hospital due to unconsciousness with limb twitching for 15 d. He had experienced intermittent abdominal pain and had not sought regular medical attention. The patient reported that intermittent abdominal pain first appeared approximately 3 months prior to admission, with episodes occurring 2-3 times per week (each lasting 2–4 h) and no obvious triggers. Proximal muscle weakness gradually developed 1 month after the onset of abdominal pain, starting with difficulty lifting arms, which worsened over time. Encephalopathic symptoms (unconsciousness and limb twitching) emerged 15 days before admission, coinciding with increased frequency of abdominal pain (daily episodes) during this period. On admission, he was found to have hypertension (178/94 mmHg), and his weight had decreased from 80 kg 6 months earlier to 65 kg at the time of admission. The weight loss was attributed to reduced food intake due to recurrent abdominal pain, leading to chronic energy deficiency. Further inquiry revealed no history of alcohol consumption, use of porphyrogenic medications (e.g., barbiturates and sulfonamides) prior to onset, or recent infections. The patient reported work-related stress 1 month before the exacerbation of symptoms, which may have contributed to the acute attack. On examination, he was found to be emaciated. The supraspinatus, infraspinatus, and deltoid muscles were atrophied (Figure 1). On motor examination, muscle weakness showed a proximal-dominant pattern: proximal upper limbs (deltoid, biceps brachii) had Grade 2 strength (unable to overcome gravity during arm elevation); distal upper limbs (wrist extensors and finger flexors) had Grade 3 strength (limited movement against mild resistance); lower limbs had Grade 4 strength (slightly weakened hip flexors/knee extensors but sufficient for independent ambulation). Upper limb muscle tone was reduced (consistent with the atrophy), with diminished biceps/triceps reflexes (1+); lower limb patellar/Achilles reflexes were normal (2+). No other abnormalities were noted on physical examination. Electromyography revealed neurogenic damage, with reduced motor unit potential amplitude and increased polyphasic potentials in the atrophied deltoid and supraspinatus muscles, indicating denervation-related weakness. Nerve conduction studies were also performed, showing normal sensory nerve action potentials (SNAPs) in the median and ulnar nerves, but mild reductions in motor nerve conduction velocities (MNCVs) of the axillary (42 m/s, reference range: 45–60 m/s) and musculocutaneous nerves (40 m/s, reference range: 43–58 m/s), consistent with axonal involvement in proximal motor nerves. In addition, cervical spine MRI (March 2, 2024) demonstrated no intervertebral disc herniation, spinal canal stenosis, or cord compression. Brachial plexus MRI showed no abnormal signal intensity or structural lesions in the bilateral plexuses, excluding root or plexus compression. On sensory examination, the patient reported no deficits in light touch, pinprick, or vibration sense in the upper or lower limbs, and no hyperesthesia was observed. On February 26, 2024, an out-of-hospital cranial MRI showed multiple abnormal signal shadows in bilateral parieto-occipital cortical areas and bilateral cerebellar hemispheres, with low or equal signals in T1WI and high signals in T2WI and FLAIR. At the time of this initial MRI, the patient presented with persistent unconsciousness episodes (3-4 times daily) accompanied by limb twitching, ongoing abdominal pain (daily episodes), and proximal muscle weakness (proximal upper limb strength Grade 2). Laboratory tests revealed serum sodium (S-Na) of 132 mmol/L (reference range: 135–145 mmol/L), blood pressure of 185/98 mmHg, and serum alanine transaminase (S-ALT) of 45 U/L (reference range: 5–40 U/L). On March 15, 2024, a repeat cranial MRI in our hospital indicated that the abnormal signals had essentially disappeared, as shown in Figure 2. At the time of the follow-up MRI, the patient's consciousness had returned to normal, with no recurrence of seizures or abdominal pain; proximal upper limb strength improved to Grade 3. Repeat tests showed S-Na of 138 mmol/L, blood pressure of 135/85 mmHg, and S-ALT of 32 U/L. Given that the patient presented with unexplained persistent abdominal pain and had epilepsy as the initial symptom, porphyria could not be ruled out. Therefore, we collected the patient's urine and exposed it to sunlight, the photooxidation of accumulated porphyrin precursors, specifically PBG and δ-ALA, which are inherently unstable under light, which showed that the urine appeared wine-colored (see Figure 3).The test for PBG in the urine was positive, and the mutation sites identified by genetic testing are shown in Figure 4 and Table 1. This indicates that the patient has a heterozygous c.445C > T mutation in the HMBS gene. This mutation is a splicing mutation, which leads to a functional defect in HMBS (PBGD) and thereby induces AIP. Combining the patient's clinical symptoms (epileptic seizures, intermittent abdominal pain, and muscle atrophy), imaging findings (reversible abnormal signals on cranial MRI), and the detection of a heterozygous c.445C > T mutation in the HMBS gene, our hospital definitively diagnosed AIP complicated by PRES. After diagnosis, the patient was referred to another hospital for further treatment. The specific regimen included the following: Intravenous hemin administration (4 mg/kg once daily for 7 consecutive days) to inhibit δ-ALA synthase (ALAS) activity and reduce porphyrin precursor accumulation; discontinuation of sodium valproate (which may exacerbate AIP) and replacement with levetiracetam (500 mg twice daily) for seizure control; and maintenance of acid-base balance, correction of electrolyte disturbances, and a high-carbohydrate diet (daily intake ≥ 300 g) to stabilize metabolism. Follow-up at 1 month after discharge: The patient gained 5 kg in weight, muscle strength of both upper limbs recovered to Grade 3, with no recurrence of epileptic seizures or abdominal pain, and urinary PBG qualitative testing was negative. Follow-up at 2 months: Weight recovered to 75 kg, muscle strength of all limbs returned to Grade 5, and the patient had resumed normal work and daily life.

## 3. Discussion

AIP is a rare hereditary metabolic disorder, one of the porphyrin metabolic disorders. AIP is caused by a mutation in the HMBS gene (also known as PBGD) and is involved in the synthesis of porphyrinogen in the porphyrin biosynthesis pathway, with a prevalence rate of 50 cases per 1 million people [2–4].

In this case, the patient presented initially with epilepsy, accompanied by PRES. It is relatively rare for AIP patients to present epilepsy as the first symptom. Epilepsy is a neurological disorder, with symptoms that differ from those of AIP, such as abdominal pain and neuropsychiatric symptoms. The presence of epilepsy in patients with AIP may indicate a broader neurological impact, requiring further detailed neurological evaluation and monitoring. The patient also presented with PRES, an uncommon clinical syndrome characterized by reversible changes in the posterior white matter of the brain, and often associated with hypertension, immunosuppressive therapy, or other causes [5]. The pathogenesis of PRES is still unclear, and there are several proposed hypotheses for its pathogenesis, including high perfusion pressure breakthrough and vascular endothelial injury. It is generally considered a result of a combination of factors, such as increased activity of the renin–angiotensin system, increased blood pressure, water–sodium retention, metabolite accumulation, and damage to the blood–brain barrier. The occurrence of PRES in AIP patients may be due to the abnormal accumulation of porphyrin metabolites in the body caused by AIP, causing cardiovascular spasm, contraction, and other abnormal manifestations, triggering vasogenic edema and leading to the onset of PRES [6]. The occurrence of epilepsy with PRES in AIP patients is relatively rare, suggesting that the neurological effects of AIP may be more complex. In this case, comprehensive evaluation and treatment were needed to improve the prognosis of the patient. The patient was treated with sodium valproate out-of-hospital after the onset of epilepsy, which is often not recommended for treating AIP and may exacerbate the patient's condition. Vivek et al. reported a case of AIP presenting with nonconvulsive status epilepticus and PRES, which shares the rare comorbidity of AIP and PRES with our case. However, distinct differences exist: our patient is a young male (28 years old) with epilepsy as the initial symptom, whereas their case primarily manifested as nonconvulsive status epilepticus. This highlights the heterogeneous neurological presentations of AIP, expanding the clinical spectrum of its initial symptoms. Furthermore, our case provides more comprehensive clinical data, including detailed genetic testing sequential imaging changes (resolution of MRI abnormalities), and long-term follow-up outcomes, which strengthen the diagnostic and prognostic evidence for such rare comorbidities. The treatment strategy for our patient, including intravenous hemin administration, discontinuation of sodium valproate (a known AIP exacerbant), and initiation of a high-carbohydrate diet, is consistent with the recommendations in the Chinese Expert Consensus on Diagnosis and Treatment of AIP. Specifically, early use of hemin is emphasized in the consensus to inhibit ALAS activity, rapidly reduce porphyrin precursor accumulation (PBG and δ-ALA), and alleviate symptoms [7]. Our case further validates this approach, as the patient showed significant improvement after hemin therapy. In addition, the replacement of sodium valproate with levetiracetam aligns with the consensus's warning against porphyrogenic antiepileptics, demonstrating the practical value of adhering to guideline-based treatment in improving patient outcomes.

This case highlights the heterogeneous neurological manifestations of AIP, particularly the rare presentation of epilepsy as the initial symptom combined with PRES. It underscores the importance of considering AIP in the differential diagnosis of cryptogenic epilepsy with unexplained abdominal pain and emphasizes early genetic testing (e.g., HMBS mutation analysis) and adherence to guideline-based management (e.g., avoiding porphyrogenic drugs such as sodium valproate and early hemin administration) to improve prognosis. In addition, the comprehensive follow-up data in this case reinforce that long-term supportive care (e.g., high-carbohydrate diet) plays a crucial role in maintaining remission [8]. Future studies may further explore the correlation between specific HMBS mutations and PRES, as well as the potential value of Traditional Chinese Medicine in AIP treatment, to refine the management of this rare disease.

## Figures and Tables

**Table 1:** Genetic test results of the patient.

Gene	Chromosome coordinate	Variation point	Gene subregion	Zygotic state
HMBS NM_000190.3	chr11: 118,960,922	c.445C > T p.Arg149^∗^	Exon 8	Heterozygosis

^∗^Stop codon.

## Data Availability

The data are available on request from the corresponding author.

## References

[1] Anyaegbu E., Goodman M., Ahn S. Y., Thangarajh M., Wong M., Shinawi M. (2012). Acute Intermittent Porphyria: A Diagnostic Challenge. *Journal of Child Neurology*.

[2] Spiritos Z., Salvador S., Mosquera D., Wilder J. (2019). Acute Intermittent Porphyria: Current Perspectives and Case Presentation. *Therapeutics and Clinical Risk Management*.

[3] Kumar V., Lalawmpuia C., Lodhi P., Guglani B. (2023). Acute Intermittent Porphyria Presenting With Non-Convulsive Status Epilepticus and Posterior Reversible Encephalopathy Syndrome. *Neurology India*.

[4] Stein P., Badminton M., Barth J., Rees D., Stewart M. (2013). Best Practice Guidelines on Clinical Management of Acute Attacks of Porphyria and Their Complications. *Annals of Clinical Biochemistry: International Journal of Laboratory Medicine*.

[5] Zhang B., Bu C., Zhao Y., Xia Z. (2024). Acute Intermittent Porphyria Presenting With Posterior Reversible Encephalopathy Syndrome, Reversible Cerebral Vasoconstriction Syndrome and Myocardial Ischemia: A Case Report and Review. *Psychology Research and Behavior Management*.

[6] Li Yang A., Ma L. M., Zhang H. J., Zhang J. W. (2022). Novel Mutation of Hydroxymethylbilane Synthase in a Case of Acute Intermittent Porphyria Presenting With Posterior Reversible Encephalopathy Syndrome. *Journal of College of Physicians and Surgeons Pakistan*.

[7] Zhang S., Zhang S., Liu M. (2025). Expert Consensus on Diagnosis and Treatment of Acute Intermittent Porphyria in China (2024 Edition). *Journal of Rare Diseases*.

[8] Schmid R., Schwartz S., Watson C. J. (1954). Porphyrin Content of Bone Marrow and Liver in the Various Forms of Porphyria. *Medcine*.

